# Potentials and barriers of digital patient education in rheumatic disease management: an exploratory qualitative interview study

**DOI:** 10.1007/s00296-025-05893-5

**Published:** 2025-06-04

**Authors:** Franziska Lumma, Johannes Knitza, Felix Mühlensiepen

**Affiliations:** 1 Medicstream, Hürth, Germany; 2 Institute for Digital Medicine, University Hospital Giessen-Marburg, Philipps University, Marburg, Germany; 3 Center for Health Services Research, Faculty of Health Sciences Brandenburg, Brandenburg Medical School Theodor Fontane Seebad 82/83 Rüdersdorf bei, 15562 Berlin, Germany; 4 Université Grenoble Alpes, AGEIS, Grenoble, France

**Keywords:** Digital patient education, Rheumatic disease management, Digital health, Chronic disease, Self-management, Health literacy

## Abstract

Patient education (PE) is essential, however hindered by the limited time of rheumatology healthcare professionals. Digital patient education (DPE) promises to bridge this care gap. This study explores the potential and challenges of DPE in rheumatology. Semi-structured expert interviews were conducted with 14 stakeholders across healthcare levels, including rheumatologists, specialized nurses, patient representatives, policymakers, and industry experts. Data were analyzed using qualitative content analysis following Kuckartz’s methodology, with intracoder reliability assessed via the Brennan-Prediger Kappa coefficient. Three main themes emerged in the qualitative content analysis: (1) the status quo of PE in general, (2) the potentials and challenges of DPE and (3) DiRhIS as an emerging tool in the field of DPE in rheumatology. Participants highlighted significant gaps in traditional PE, including limited consultation time, inadequate access to information, and misinformation risks. While DPE was recognized as a promising tool to enhance patient knowledge, adherence, and shared decision-making, systemic barriers hinder adoption. Experts identified key obstacles such as resistance among healthcare providers, limited digital literacy among patients, and challenges in integrating DPE into clinical workflows. A hybrid model combining digital and in-person education was favored to address patient needs across different literacy levels. Successful implementation requires structured integration, stakeholder involvement, and strategies to address infrastructural and attitudinal barriers. Digital patient education (DPE) has the potential to enhance accessibility and the quality of patient education in rheumatology. Findings emphasize the need for personalized patient education, where the balance between face-to-face and digital learning is adapted to individual preferences, health literacy levels, and accessibility needs.

**Supplementary Information:**

The online version contains supplementary material available at 10.1007/s00296-025-05893-5.

## Introduction

Patient education (PE) plays a vital role in rheumatology management [1] by empowering patients to actively participate in their treatment and take proactive measures to mitigate disease progression [2]. However, studies indicate a substantial gap in the quality and accessibility of PE, with only 8% highly satisfied and 17% satisfied with available information [3]. This gap is further pronounced among patients with lower health literacy, older age, and higher disease activity [4], highlighting the necessity for tailored, accessible, and evidence-based educational resources.

Digital health technologies present a transformative opportunity to enhance PE and care delivery in rheumatology [5, 6]. Digital patient education (DPE) has demonstrated efficacy in improving self-management, treatment adherence, and overall disease outcomes [7, 8]. The European League Against Rheumatism (EULAR) has underscored the necessity for PE to be an interactive process, personalized, and seamlessly integrated into routine care [1]. In alignment with these principles, individualized and integrated DPE offers continuous, on-demand access to reliable information, customized to meet the specific needs of each patient [9]. Preliminary findings indicate a strong willingness among patients to engage with digital solutions in PE [3, 9]. A majority of rheumatic patients (89%) reported being very likely, likely, or rather likely to use digital educational tools, and 82% of specialized rheumatology nurses (SRN) would welcome DPE as well. The patient-perceived advantages include improved access (90%), geographic independence (73%), and access to information that is otherwise difficult to obtain (57%) [3]. Despite the potential benefits of DPE, adoption remains limited. While physicians recognize their value, they hesitate to integrate them into routine practice [10]. In fact, 85% of doctors in 2021 did not provide their patients with digital personalized information [9]. Barriers to implementation include technological infrastructure challenges, concerns about increased workload, and resistance to changes in traditional patient-provider interactions [11, 12]. Moreover, concerns regarding misinformation, data security, and the absence of human interaction have been identified as impediments to the broader adoption of DPE solutions [3].

In April 2024, the “Digital Rheumatology Information System” (DiRhIS) was made available to rheumatologists. This joint project of the German Rheumatology Association (BDRh) and medicstream aims to enable healthcare providers to share validated and low-threshold health information with patients before, during, or after consultations. DiRhIS provides healthcare professionals with the flexibility to distribute pre-established playlists of general health information or to curate content individualized from a comprehensive database. A multidisciplinary quality board oversees the accuracy and high standards of the content. This validated information is shared digitally via link or QR code [13].

A qualitative study with semi-structured stakeholder interviews was conducted to identify the barriers and facilitators of DPE in German rheumatology care. By focusing on the newly introduced DiRhIS system, the study contributes to the growing body of knowledge around digital health adoption in clinical practice. In doing so, it seeks to understand how DPE tools are received by a diverse group of stakeholders, ranging from rheumatologists and SRN to patient representatives, policy maker, and digital health experts.

## Methods

To explore the perceived challenges and opportunities of DPE in rheumatology, an exploratory qualitative study was conducted using semi-structured stakeholder interviews.

### Participants and sampling

Participants were identified through a systematic analysis and classification of stakeholders involved in digital education in rheumatology, ensuring the integration of diverse perspectives and enabling a multi-level approach. Given the exploratory nature of the research design, a criteria-driven and purposeful sampling strategy was employed. This approach allowed for the theoretical definition of sampling criteria within the quota plan, ensuring a systematic and non-random selection of interview participants with a focus on the rheumatology sector [14]. Participants were contacted via telephone, email, LinkedIn, or in-person.

### Data collection and analysis

The interviews were conducted between May and July of 2024 using a semi-structured interview guide. The guide focused on three overarching themes: (1) the current state of PE in rheumatology, (2) perceived potentials and challenges of DPE, and (3) the specific role, implementation, and future potential of DiRhIS. Themes 4 (sustainability) and 5 (future visions) are not part of this publication’s scope.

To ensure clarity, engagement, and the collection of pertinent data, the interview questions were developed using Helfferich’s SPSS principle, which stands for “collect, test, sort, and summarize” [15]. The interview guide was developed to explore the contextual understanding, perceived challenges, and opportunities related to the implementation of DiRhIS and DPE. While it was not based on specific previous interviews or guidelines, it was informed by current discourse in digital health. For instance, Knudsen et al. [12] highlighted the value of hybrid models in DPE, prompting questions about the feasibility of fully digital formats. Following an initial draft, two rounds of revision were conducted after cognitive pretesting with a layperson and a non-rheumatology healthcare expert, using verbal probing and think-aloud techniques. Feedback led to refinements in structure, language, and the removal of irrelevant questions. The final version is provided in Supplementary Material 1.

The interviews were conducted remotely via the video conferencing platform Microsoft Teams by F.L., with the interviewer and interview partner participating via this medium exclusively. To establish rapport, the interviews commenced with an explanation of the study, a brief well-being check and personal exchanges, to facilitate participant openness and reduce potential biases. Data collection adhered to ethical standards, with informed consent obtained prior to recording and transcription [15]. No socio-demographic data was collected, and no financial incentives were provided to the participants. During the interviews, brief notes were taken to facilitate follow-up questions.

For analysis, audio recordings were transcribed verbatim following a defined transcription protocol that emphasized textual content over non-verbal cues. An inductive qualitative content analysis, guided by Kuckartz’s methodology, was employed by F.L. and F.M. to systematically explore emergent themes [16]. Initial coding developed thematic categories from the data, followed by iterative refinement into subcategories to enhance depth and accuracy. Data reliability was ensured through intracoder-reliability testing, employing the Brennan-Prediger Kappa coefficient in MAXQDA [17].

Representative quotes from the transcripts were selected, translated into English and incorporated into the manuscript, which was compiled in accordance with the Consolidated Criteria for Reporting Qualitative Research (COREQ) [18].

## Results

### Intracoder-reliability

The Intracoder’s reliability was evaluated using observed agreement of 0.85 (85%) and random agreement of 0.02 (2%), yielding a kappa coefficient of 0.84, indicating strong agreement (84%) between the two coding rounds.

### Participant characteristics

14 representative stakeholders (*N* = 14) were interviewed. A summary of the interviewees, including their roles, is provided in Table 1. The mean interview duration was 43 min. Notably, the two representatives of patient organizations who were interviewed did so as private individuals and not in their official capacity for the respective organization. Three interviews (R2-mi, BMG-ma, and P-ma) were subject to time constraints. The sample comprised of five representatives from the micro level (*n* = 5), four from the meso level (*n* = 4), and five from the macro level (*n* = 5). The planned quota of three SRNs was not fully achieved due to the short-notice cancellation of one SRN interview.

**Table 1:** Participant characteristics

Level	Field	Code	Position	Duration
Micro	Rheumatologist	R1-mi	Pediatric Rheumatologist, Member of the professional association board	37 m 28s
	Rheumatologist	RR-mi	Head of Rheumatology in a clinic, active in the Digital Rheumatology Network	47 m 58s
	Rheumatologist	R2-mi	Rheumatologist, Member of the Professional Association	22 m 13s
	SRN	SRN1-mi	SRN, Member of the Professional Association	49 m 332
	SRN	SRN2-mi	SRN und practice manager	43 m 29s
Meso	Patient Advocacy	PR1-me	Rheumatic patient, active in patient advocacy	1 h 23 m 39s
	Patient Advocacy	PR2-me	Research partner in patient advocacy with extensive knowledge in PE	1 h 12 m 38s
	Patient & Start-up	RPF-me	Rheumatic patient, Co-founder of a Rheumatology App for PE	39 m 52s
	Pharma	P1-me	DiRhIS Sponsor, Pharma representative	41 m 28s
Makro	Health-Tech Consultant	SB-ma	Founder in Gender Medicine and digital health product development and consulting	47 m 40s
	Health Insurance	HI-ma	Digital Health Expert, Health Insurance Start-up scout	40 m 28s
	Research	ER-ma	Project Manager, Bertelsmann Foundation Health Ecosystems	45 m 35s
	Policy	BMG-ma	Specialist, Sustainability and Health Competence, Federal Ministry of Health	57 m 17s
	Pharmaceutical industry	P-ma	Deputy Head of Rare Diseases and Neurology with digital health solutions competencies	22 m 7s
			⌀	43 m 29s

### Themes

Three main themes emerged in the qualitative content analysis: (1) the status quo of PE in general, (2) the potentials and challenges of DPE (3) DiRhIS as an emerging tool in the field of DPE in rheumatology.

### The status quo of patient education

Participants provided diverse perspectives on the current state of PE, highlighting both strengths and areas for improvement. Some experts were positive, emphasizing the individualized attention patients receive in specialties such as rheumatology.

“I don’t think all rheumatologists are equally good, but overall, I think patients are well informed.” (P1-me, pos. 53–63).

“I think a lot of things are going well for us. A lot of things vary from person to person, just like in other professions. If someone puts in more effort or has more knowledge, it’s a little better than in other cases.” (R1-mi, pos. 147–149).

However, the majority identified significant areas for improvement. Experts frequently criticized the limited time allotted for patient consultations, often cited as about seven minutes. They described this as:

“(…) a disaster, because no human being can really open up in that period of time.” (ER-ma, pos. 194–195).

“In seven minutes, it is not possible to fully and sufficiently inform even the most knowledgeable person about a disease. (…) Especially the things that patients need to do proactively, such as nutrition, stress management and compliance. These are things that need a certain amount of time, they also need a certain amount of trust between the doctor and the patient and that is not possible in seven minutes.” (SB-ma, pos. 97–105).

Another interviewee noted that often a lack of empathy and structure, as well as poor health literacy and communication, can cause confusion and disorientation for patients:

“But there is often a lack of empathy, a lack of clear structure, a lack of explanation. People who have lower health literacy and are uncertain or are in a life-changing journey, and that’s a very personal assessment of when something is life changing. They don’t just have questions at the moment of the consultation, but also afterwards and sometimes even before. There is often a question with communication, a lot of disorientation.” (HI-ma, pos. 184–189).

Further critique has been directed towards the insufficient consideration of the relationship between promoting self-efficacy through PE and treatment success in healthcare.

“It is scientifically proven that the self-efficacy generated through patient education has value in the healthcare system, but no value is assigned to it because no one pays for it. And because no one makes direct money from it, it is viewed in the short term. Since no monetary revenue is attributed to it, it is disregarded, even though it is actually important for outcomes. Especially for diseases that consume many resources in the healthcare system, like diabetes, cardiovascular health, cancer, or rheumatism.” (SB-ma, pos. 128–134).

### Trust and scepticism

The personal relationship between physician and patient was emphasized as a cornerstone of effective PE and trust-building, particularly in the context of chronic illness:

“For me, the doctor-patient relationship is extremely important, especially when dealing with a chronic condition where a high level of mutual trust is necessary.” (PR1-me, pos. 163–164).

It was hypothesized that the presence of trust in a physician has a significant impact on patients’ propensity to engage in online research. One participant noted:

“If I, as a patient, trust my doctor and he provides me with the information he believes is right for me, I think people are less likely to search the internet. However, if I don’t fully trust my doctor or tend to believe I know better, I will likely continue searching online regardless.” (P1-me, pos. 88–93).

Beyond that, concerns were raised about reliance on Internet-based information, with several warning of the risks of misinformation:

“I sometimes have concerns when patients simply research things on the internet - not that the patient should not be free to explore everything openly - but the risk of coming across misinformation or ‘half-truths,’ so to speak, is simply very high.” (SRN1-mi, pos. 114–120).

“The major risk in patient education, ever since the invention of the internet – essentially since the invention of Google - is that patients independently search for information when they are hit with a diagnosis, without the doctor explaining what it truly means for them or discussing alternative treatment options. Then ‘Dr. Google’ comes into play, and things get critical online. We once ran an exercise on this. You can find astonishing things on the internet. Back then, in cardiology, we came across references to a ‘heartworm’ that supposedly causes atrial fibrillation.” (P-ma, pos. 77–84).

### Potentials and challenges of digital patient education

In the domain of digitalization, experts concurred that German health care system is encountering challenges:

“It’s complicated and involves a lot of data protection issues. It’s not a sure-fire success.” (BMG-ma, pos. 186–187).

“Many people think: Oh God, computers are so difficult, I am doing something wrong.” (PV1-me, pos.119–120),

“It has to be said that the infrastructure simply doesn’t work. Especially here, where we are based, the internet is regularly down. That causes us major problems.” (R2-mi, pos. 22–23).

Notwithstanding, the integration of digital tools into PE was recognized to offer significant potential in enhancing efficiency and promoting self-management. Participants highlighted the benefits of digital education in reducing resource consumption by enabling patients to better understand and manage their conditions, thereby reducing reliance on medical staff:

“I am by no means saying that visits to the doctor are unnecessary! But I can achieve better prevention with education, can ensure that we don’t run to the emergency room with a cold, to put it bluntly.” (PR1-me, pos. 485–487).

“Of course, there is also the possibility of not having extensive consultations, but of referring to certain aspects digitally. In other words, you can just say, ‘I’m going to give you basic information and then more detailed information. And they can go home and read it again and discuss it the next time’. I think that would be very helpful.” (SRN1-mi, pos. 81–86).

### Hybrid versus fully automated

The findings emphasize the preference for a hybrid model of PE that integrates digital tools with human interaction.

“(…) that as a patient you know you can still talk to a person if you have questions. (…)

You must assume that the sick patient is a bit older and not so digitally savvy. That would also help a lot with acceptance, because you know that at the end, I can still call Nurse Stephanie, and she’ll answer a question after I’ve looked at everything and maybe discuss what I’ve seen with me again. That there is a loop where there is also a human being. In other words, a human contact that supports digital patient communication.” (SB-ma, pos. 147–156).

“I don’t need a doctor just to impart knowledge. I need him for coordinating/changing medications, discussing and evaluating blood values. When it comes to pure knowledge, I can imagine it. Otherwise, I tend to be very empathetic and need a counterpart simply because I really appreciate facial expressions, body language, and a pleasant atmosphere. They give me confidence in the treatment.” (PR1-me, pos. 516–520).

Others acknowledged the need for a dual approach in PE, as not all patients are digitally literate. Emphasizing that, ideally, digital solutions should minimize human interaction, especially for tech-savvy generations.

“So I think the answer is it depends. I think there are simply people who just want very efficient instructions, education via an app. They want the medication sent to their home, they only want to go to the doctor twice a year and maybe have a brief discussion, and there are others, older people in particular, who are alone and some don’t understand it so quickly, they prefer a face-to-face consultation or education. So I very much believe that it depends.” (RF-ma, pos. 122–128).

“The maxim is actually: as little human interaction as possible.” (HI-ma, pos. 222–223).

### DiRhIS as an emerging tool in the field of digital patient education in rheumatology

In the context of increasing interest in DPE, DiRhIS represents a recent initiative designed to enhance information delivery in rheumatology care through a structured, accessible, and patient-centred digital platform.

### Enhancing patient education and personalized care

The merits of DiRhIS are frequently enumerated, including its contributions to PE, its influence on health literacy, and its promotion of shared decision making.

“(…) probably the knowledge, the prior knowledge (improves). Thus, possibly the compliance. And perhaps the quality too.” (RR-mi, pos. 155–156).

“And because the patient can express himself or herself better, you can diagnose better and make better treatment decisions together with the patient.” (SB-ma, pos. 170–172).

“So I think it can definitely significantly improve targeted, comprehensive education.” (R2-mi, pos. 105–106).

The interviewees elucidated that DiRhIS boasts the advantage of patient-centricity and flexibility, where rheumatologists can personalize information packages according to the individual needs of the patient.

“This can help the patient not to be overloaded with information, but to get an information package that is tailored to their needs (…).” (P1-me, pos. 125–128).

“Then I can get reliable information about my disease, have a knowledge package tailored to me, and know that I don’t have to deal with the rest of the digital world to find the right information.” (PR1-me, pos. 263–265).

### Complementary role

Experts highlighted DiRhIS ability to integrate seamlessly into care pathways, providing a centralized platform for managing PE. As remarked:

“You have a process integrated into the care pathway, so to speak, which can also be used to manage patients.“ (RPF-me, pos. 127–128).

However, consensus emerged among study participants, with many underscoring DiRhIS’s role as a complementary modality to in-person consultations.

“I find it rather complementary, so what I’ve seen, I’ll say again, it’s a very good tool to use in a complementary way.” (SRN2-mi, pos. 222–223).

### Practical and psychological accessibility

Reduced logistical burden and the convenience of readily accessible on-demand educational materials were also noted by one participant:

“(…) and it’s always available. I no longer have to keep as many materials in cabinets behind me. Currently, I always have the right material. For example, if the right one is not copied, the patient can’t see it because you don’t have time. (R2-mi, pos. 100–104)

Psychological accessibility, particularly for patients managing complex conditions, was another seen advantage, as it enables patients and their families to revisit information at their own pace.

“I think that’s a very important factor, especially when you think about older patients, for example, that they have the opportunity to look at it again in peace and quiet at home with their family members. This is particularly helpful when it is a really serious diagnosis.” (HI-ma, pos. 162–165).

### Systemic barriers

The most frequently mentioned challenge of the DiRhIS project is its integration into daily practice. It was also regarded as necessitating a dedicated effort to overcome existing capacity constraints and align workflows, particularly in already strained practices:

“But the challenge, I think, will be to get it into the practices, into the daily routine, that someone is there. In other words, finding someone who has the time to do this with the patient. And that people familiarize themselves with the program so that they know the content well. And that, if I may say so, is where I see a bit of a difficulty at the moment, because it’s full everywhere. It’s always very tight everywhere.” (SRN1-mi, pos. 159–164).

**“**So you have to encourage the staff. We must activate the multiplicators so that it works well. We must make everyone aware that we now have a different level. And that takes time. It doesn’t happen overnight; I’ve learned that. You also overtax the practice if you try to establish a new project in a setting where there is already a lot of upheaval.” (R2-mi, pos. 108–113).

One expert raised the issue that a crucial element of effective digital health initiatives, namely the active involvement of patients or patient organizations throughout the development and implementation phases, had not been adequately considered in the DiRhIS project.

“(…) we are only involved as information providers. So, we’re not research partners, we’re not cooperation partners. Well, I always like what happens in other things. Ultimately, nothing without us, only with us. How can you build a platform without those it is actually meant for? (…) So from that point of view, patient participation always.” (PR2-me, pos. 338–344).

### Soft barriers

The introduction of DiRhIS comes with several perceived challenges, particularly concerning soft factors such as user openness, engagement, adaptability and digital competencies. Resistance among medical staff was highlighted, as followed:

“But you also always have the hurdle that there are people who are afraid to invest the time and only see the time they would have to invest now to implement it and fail.” (P1-me, 186–188).

Conversely, the significance of patient readiness to utilize digital educational instruments and the extent of practices’ receptivity were identified as impediments to adoption:

“There has to be a certain openness on the part of patients to really want to consume it. I think that’s a challenge. I don’t know how open practices are to taking part in it” (SB-ma pos. 177–178).

Another critical factor noted was the need to capture and sustain user attention amidst increasing digital distractions:

“You always have to look at how effective something is. Putting something on display and saying it will work is increasingly proving to be fatal, because we simply have this enormous attention problem. People sometimes leave an application after just 30 seconds if they don’t immediately understand something or get what they want.” (BMG-ma pos. 469–474).

### Product-related barriers

With regard to functionality and content, the experts identified challenges and underscored the need for certain requirements, among other issues. One expert noted that difficulties may stem from a paucity of transparency regarding the review process or an excess of content.

“A disadvantage is often that it becomes difficult when I have a lot of things in there that I’ve never read myself or that I don’t even know who authorized.” (R1-mi, pos. 149–151).

With respect to the content, it was observed that the adaptation to patient-specific medical data is deficient. As articulated:

“It is not personalized, and as such, it remains general information for the time being.” (BMG-ma, pos. 428).

Another participant offered commentary on the absence of interactive elements:

“So it is quite nice. But its impact is probably limited because it’s not interactive. If there was a chat functionality in there, for example.” (RF-mi, pos. 164–165).

### Synthesis

**Fig. 1:**
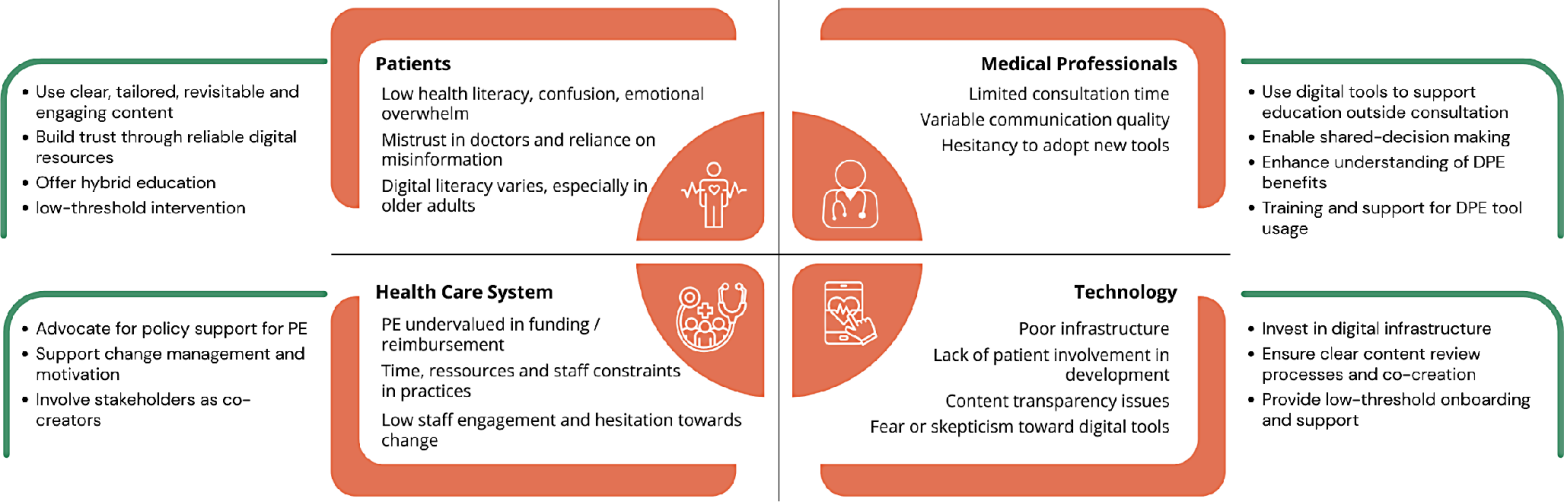
Perceived barriers and potential solutions for DPE implementation in rheumatology care

The interview findings can be synthesised into two key areas: perceived barriers and potential solutions for DPE implementation in rheumatology care (Fig. 1).

## Discussion

Face-to-face interactions and in-person education remain fundamental. Yet, the findings of this study elucidated significant gaps of PE in rheumatology, particularly with regard to time constraints, accessibility, and the integration of promising digital solutions. Consultations with an average duration of just seven minutes, have been described by stakeholders as insufficient for addressing complex self-management issues and new diagnoses. One expert emphasized that despite its proven impact on adherence, disease progression, and healthcare utilization [19], PE remains critically undervalued in clinical practice. This aligns with findings from Becker et al. [3], who found that only 25% of patients rated their disease education as satisfactory, with family planning and disease coping identified as the most pressing unmet information needs. While some experts noted that consultation times vary across specialties – rheumatology consultations averaged 15 min in a 2021 study [20] – the projected rise in rheumatic and musculoskeletal disease cases and the growing shortage of rheumatologists [6, 21, 22] imply a further decline of consultation duration, exacerbating existing challenges in effective PE delivery.

Another critical insight from our study is the role of trust in shaping health information-seeking behaviours. Experts hypothesized that patients with lower trust in their physicians are more likely to seek independent information online, exposing them to both credible resources and misinformation. In the post-COVID-19 era, the risks of “infodemics”, where misinformation fuels confusion and distrust in healthcare authorities, have been well-documented [23]. Vulnerable populations are disproportionately affected, exacerbating health inequities [24, 25]. While medical professionals play a key role in countering misinformation, their ability to do so is often limited by time and resource constraints. The interviews revealed that addressing misinformation can be a time-consuming and exhausting process for healthcare providers, especially when patients strongly trust inaccurate online content. This underscores the significant burden that unreliable information places on clinical encounters. Results by Becker et al. are in line with this, revealing that 70% of patients use the internet as their primary source of information. Noteworthy is, that only 6% of SRNs endorse this practice [3].

Integrating validated DPE tools into routine care is a promising strategy, as emphasized by both the literature and our interviewees [3, 12, 23]. Our findings reveal conflicting perspectives on its approach. Experts generally favoured a hybrid model – blending digital tools with face-to-face interactions – as a way to improve accessibility while maintaining the relational aspects of care. However, opinions diverged on whether DPE can fully replace in-person education. Some participants advocated for a digital-first or digital-only model to accommodate patients who prefer minimal human interaction, while others cautioned against an overreliance on technology. A recent study indicates that hybrid models, combining remote monitoring with clinician-endorsed video education, could enhance adherence, patient empowerment, and efficiency while reducing misinformation [26]. Deshpande et al. [27] similarly underscore the promise of video-based education in chronic illness management, while cautioning that it must be supplemented by human support. Concurrently, Knudsen et al. [28] indicated that patients often require increased support from medical staff after engaging with e-learning, particularly in the early stages of their disease. In consonance with preceding research, the risk of digital exclusion, particularly among older patients with limited technological skills or access to digital infrastructure, is stated as a salient concern [29]. These findings reinforce the necessity of a tailored, inclusive approach to DPE that accounts for varying patient needs. Comparably Clemensen et al. suggests for telehealth solutions a participatory approach, emphasising mutual learning and co-creation as key principles [30]. The EULAR guidelines advocate for the active involvement of patient representatives throughout all phases of the design, implementation and evaluation process of interventions [1]. Comparative studies will be essential to establish evidence for the most effective PE methods. The recent publication of disease knowledge questionnaires provides objective outcome measures to support such research [31, 32].

Similarly, DiRhIS was viewed by experts as a promising complement to existing PE efforts, especially in improving health literacy and patient-centred care. By providing structured, validated educational content, DiRhIS has the potential to counter misinformation and alleviate the burden of unstructured online searches. Despite the potential, several implementation barriers persist. Experts highlighted besides missing product features and requirements, systemic barriers, such as inadequate technological infrastructure and resistance among healthcare professionals, often stemming from concerns over an amplified workload and unclear benefits.

In comparison with preceding publications on DPE, the present study offers a novel contribution by examining stakeholder perspectives on a specific DPE system, DiRhIS, shortly after its national rollout. The present study provides a multi-level qualitative analysis of the broader implementation context, integrating voices from rheumatological professionals, patient representatives, industry, and policy sectors. The broad scope of the study enriches the understanding of system-wide potentials, facilitators and barriers, particularly within the German rheumatology setting. This is the first qualitative study to address DiRhIS and its integration into rheumatology care in the real world.

For clinicians, this study provides insights into how DPE can be leveraged to optimize consultation time and enhance patient empowerment. For researchers, the qualitative findings highlight implementation challenges, illuminate contextual factors affecting adoption, and serve as a foundation for future evaluations of DPE efficacy and integration strategies. Future research should quantify the impact of DPE versus traditional formats by assessing time efficiency, health literacy, clinical outcomes, and benefits in resource management. Additionally, identifying integration enablers and developing transferable implementation strategies could guide broader digital health adoption. Implications for the global rheumatology community include the need to co-develop scalable, evidence-based DPE strategies that respond to clinician constraints, digital health literacy gaps, and patient needs.

Despite the novelty of our approach, the study has several limitations. First, the sample size, while appropriate for qualitative inquiry, remains relatively small and may limit the generalizability of findings. The purposive sampling approach may also introduce selection bias, as participants may not fully reflect the broader rheumatology care landscape. Second, familiarity with DiRhIS among some participants may have contributed to positive response bias, potentially influencing the depth or favourability of their feedback. Third, social desirability bias cannot be ruled out and may have led participants to emphasize benefits over potential drawbacks of DPE. However, the inclusion of respondents without prior exposure to DiRhIS, comprising half of the sample, helped to counterbalance these effects and supports the credibility and richness of the thematic analysis.

## Conclusion

This study highlights the widening care gap in rheumatology and the increasing challenges in delivering effective PE. While DPE presents a promising opportunity to enhance patient knowledge and self-management, its fruitful integration into routine care remains complex. Findings emphasize the need for a balanced approach that combines digital and face-to-face learning, tailored to patient needs. Ensuring equitable access to high-quality, evidence-based education will be key to improving patient outcomes and addressing disparities in rheumatology care.

## Electronic supplementary material

Below is the link to the electronic supplementary material.

Supplementary Material 1

## Data Availability

The raw data supporting the conclusions of this article will be made available by the authors upon reasonable request.

## Abbreviations

BDRh: Berufsverband Deutscher Rheumatologen Service LLC
COREQ: Consolidated Criteria for Reporting Qualitative Research
DiRhIS: Digital Rheumatological Information System
DPE: Digital Patient Education
EULAR: European League Against Rheumatism
PE: Patient Education
SMN: Specialized Medical Nurse
SRN: Specialized Rheumatology Nurse
