# Supplementary material for: Potentials and barriers of digital patient education in rheumatic disease management: an exploratory qualitative interview study
Source: Rheumatol Int. 2025 Jun 4;45(6):148. doi: 10.1007/s00296-025-05893-5 (PMC12137533; doi:10.1007/s00296-025-05893-5)
Supplement: Supplementary file 1 — Supplementary Material 1 [file 296_2025_5893_MOESM1_ESM.docx]

Title: Digitale Edukation für eine nachhaltige rheumatologische Gesundheitsversorgung (DigiEduG)

**Name**: **Date**:

_____________________________________________________________________________________________________________________________

1. **Greeting & introduction**
2. **Short explanation of the research project**
   - Objective: Survey of requirements and challenges of the introduction of digital health technologies with a focus on digital education in rheumatology, particularly on the project - Digital Rheumatological Information System (DiRhIS) of the BDRh and medicstream.
   - Framework: Case-Study of the Master's thesis in Public Health on the: ‘Sustainable integration of digital transformation processes in the healthcare sector’.
3. **Explanation of the procedure**

Start with demographic questions. Next, questions about the digitalisation of the health sector will be asked, followed by a short introduction to DiRhIS with corresponding questions. Lastly, we will look at sustainability in the health care sector.

- - Duration oft he interviwe: 30 - 45 minutes.

1. **Confidentiality and privacy policy**: digital recording and transcription of the interview; deletion after evaluation; anonymised processing; consent can be revoked at any time.
2. **Enquiry for constent**

**DiRhIS Introduction** (at the beginning of the 3rd lead topic)

The aim of DiRhIS is to enable rheumatology staff to share information with patients in a digital format.

All information displayed has been checked in advance by a quality gate (consisting of four rheumatologists and two rheumatologist specialist assistants) to ensure that the information is reliable, up-to-date and relevant.

Content can be sent that is either customised for the patient or pre-prepared information packages, a kind of playlist, can be selected and sent.

This takes the form of a QR code, an e-mail or a link. All forms of dispatch are personalised to the medical facility on request so that the solution provider is not visible to the patient.

The patient is given an information pack that may include details regarding medication, treatment options, living with the condition, and so on. These resources are available in the formats video, audio, or text.

Access requires an internet-enabled device for medical staff and patients. Medical staff need to register on DiRhIS and use the website online. Patients, on the other hand, do not need to register.

Another requirement at the moment is that users must be able to speak German, as the content is in German. However, it is planned that the content will be made available in the most common languages. Patients will be able to customise the language when they view the information on their device.

The DiRhIS project was developed within the framework of the Federal Association of German Rheumatologists and medicstream GmbH and is supported by patient representatives such as the Rheumatism League, the Association of German Rheumatologists, the specialist assistants and the specialist association Morbus Bechterews.

Abbvie is the sponsor of the project.

**Interview Guide**

| **Guiding question** | **Memo** | **Control questions** |
| --- | --- | --- |
| **1. Lead topic: Introduction** | | |
| **1.1** Please briefly explain your field of activity in the context of (rheumatological) healthcare. | - Do you have any points of interaction with rheumatological care? |  |

| **Guiding question** | **Memo** | **Control questions** |
| --- | --- | --- |
| **2. Lead Topic: Digital Transformation** | | |
| **2.1** The Bertelsmann Foundation has categorised Germany as ‘lagging far behind’ in terms of digital transformation in the German healthcare sector.  How do you explain this position? | - What is your assessment of the digitalisation of the rheumatological healthcare sector in Germany - What importance do you believe it holds? | Can you please go into more detail about __?  You rate the topic as __? Why? |
| **2.2** When you think of a successful initative/project/programme in the context of digitalization of (rheumatological) healthcare, what do you think of?    *HP Healthcare Professional*  *NP Non-Healthcare Professional* | *NP + HP:*   - What requirements do you have for digital health technology or eHealth (in rheumatological healthcare)? - In which situations do you see a clear benefit for eHealth applications (in rheumatological healthcare)?   *HP:*   - Do you recommend eHealth applications? How are your recommendations received? - Have you experienced resistance to eHealth and how did you deal with it? - Has eHealth changed the way you work? | Would you like to add something in terms of __?  When do you rate an application/feature as bad?  Do you have an example of a beneficial use case that you would like to share? |
| **2.3** What do you think about the integration of artificial intelligence (AI) into (rheumatological care) healthcare? | - What measures must be taken to ensure a successful integration of AI?   HP:   - Would you integrate AI in practice? | Can you please go into more detail about __?  You rate the topic as __? Why? |
| **2.4** How do you rate the current approach to patient education (PE)? | - What improvements could be made to PE? - Could the potential for optimization be realized through a digital format of PE? - Could a digital approach be implemented asynchronously, or would a human (synchronous) component be required? |  |

| **Guiding question** | **Memo** | **Control questions** |
| --- | --- | --- |
| **3. Lead Topic: Digital Patient Education**  Brief demonstration of DiRhIS in advance | | |
| **3.1** Were you already familiar with the DiRhIS project before the demonstration? | - Have you understood the function/task of DiRhIS? | Is something unclear? |
| **3.2** What influence do you think DiRhIS can have on the interaction between patients and healthcare professionals? | - What challenges & opportunities do you see for patients (with rheumatic diseases)? | Can you give a specific example of your assessment?  Could you please elaborate on __? |
| **3.3** How could DiRhIS be successfully integrated into (rheumatological) healthcare – and which barriers and requirements should be considered in the integration? | - How should DiRhIS (not) be financed? - What role do the various stakeholders play, such as the treatment team, patients, politicians, industry, and health insurance companies? - What is your opinion of industry involvement in the project? | Do you have an example of this?  Can you please exmaplain ___ in more detail?  So it is (not) beneficial that __ ? |

| **Guiding question** | **Memo** | **Control questions** |
| --- | --- | --- |
| **4. Lead Topic: Sustainability in healthcare** | | |
| **4.1** In 2021, the German Medical Association spoke out in favor of a climate-neutral healthcare sector by 2030?  What do you think of this? | - How do you assess this decision? | Can you please explain ___ in more detail?  Do you have an example for ___? |
| **4.2** Now coming back to DiRhIS.  Does DiRhIS harbour the potential for more sustainability in the healthcare sector? | - What role do you think digitalization plays in promoting sustainability? | What do you mean with ___.  Could you please elaborate on __- |
| **4.3** How would you define sustainability in the healthcare sector? |  | Why do you think that is?  Do you think __ should change? |

| **Guiding question** | **Memo** | **Control questions** |
| --- | --- | --- |
| **5. Lead Topic: Conclusion** | | |
| **5.1** Now please imagine healthcare in the year 2030.  How do you see this in terms of digitalization? | - Could you please elaborate on the point of health education and doctor/medical staff and patient contact? | Please explain why.  If I have understood you correctly ___.  And then? |
| **5.2** Is there anything else you would like to add to the topics discussed? |  | You said earlier that __.  Going back to the previous point __, how do you see the connection between these two points ___?  Do you remember a use case that you would like to share here? |
